# Short- and Mid-Term Prognosis of Patients Undergoing Rotational Atherectomy in Aortoostial Coronary Lesions in Left Main or Right Coronary Arteries

**DOI:** 10.1155/2019/9012787

**Published:** 2019-08-21

**Authors:** Marine Quillot, Didier Carrié, Thibault Lhermusier, Frédéric Bouisset, Romain André, Meyer Elbaz, Jérôme Roncalli, Francisco Campelo-Parada, Nicolas Boudou

**Affiliations:** Cardiology Department, University Hospital of Toulouse, Toulouse, France

## Abstract

**Objective:**

To determine short-term and mid-term prognosis in patients with calcified ostial coronary lesions who underwent rotational atherectomy (RA).

**Background:**

RA was developed to facilitate stenting in complex lesions. Treatment of calcified aortoostial coronary lesions with RA appears to have poorer procedure outcomes than nonostial lesions; yet the literature on this topic is scarce.

**Methods:**

Of 498 consecutive patients who underwent RA, a total of 80 (16.1%) presented with aortoostial lesions. A comparative, monocentric study was performed between patients with aortoostial and nonaortoostial stenosis, in a retrospective registry. The primary endpoint was the procedural success rate. Secondary endpoints were the rates of major adverse cardiac and cardiovascular events (MACE) at 30 days and 24 months.

**Results:**

The procedural success rate was high and similar in patients with and without ostial lesions (96.3%* vs* 94.7%, p=0.78), as was the rate of angiographic complications (7.5%* vs* 8.4%, p=0.80). However, the 30-day mortality rate was significantly higher in the aortoostial group (11.3%* vs* 4.8%, p=0.04), as was the 24-month rate of MACE (43.8%* vs* 31.8%, p=0.04). The aortoostial location of the lesion was an independent factor associated with the occurrence of cardiovascular events at 24 months (HR = 1.52, 95% CI, 1.03-2.26, p=0.035).

**Conclusion:**

Procedural success and complication rates were similar in patients with and without aortoostial lesions. Despite a poor short- and mid-term prognosis, rotational atherectomy appears to be a feasible and safe treatment option for calcified aortoostial coronary lesions.

## 1. Introduction

The presence of calcified coronary lesions increases the complexity of percutaneous coronary intervention (PCI) due to difficulty in stent delivery and expansion. Treatment of calcified aortoostial lesions was associated with lower success rate of balloon angioplasty procedures as compared with nonostial lesions, which led to the use of complementary devices, such as rotational atherectomy and directional atherectomy (minimally invasive treatment). Rotational atherectomy (RA) was developed in the late 1980s in order to facilitate calcified plaque debulking and was, then, the only available device to treat heavily calcified ostial coronary artery lesions. Thirty years after the introduction of the Rotablator™ system (Boston Scientific Corporation, Marlborough, MA, USA), RA remains a major technique for percutaneous coronary intervention in modern catheterization laboratories, as it is sometimes the sole method that allows a successful angioplasty procedure for complex calcified lesions. The efficacy of this device is highly operator-dependent compared with other devices, and the safety of the technique increased along with the operators' experience. However, despite its benefits, RA is only used occasionally. In the 1990's, rotablation represented 10% of all angioplasty procedures [1]. More recently, RA only constituted 3% to 5% of angioplasty procedures in high-volume centers. The characteristics of RA make it a suitable technique for specific, complex lesions, such as calcified aortoostial lesions. Previous studies showed that performing an angioplasty procedure, preceded or not by rotational atherectomy, to treat aortoostial coronary lesions results in worse procedure outcomes and in increased rates of restenosis and major cardiovascular events [2, 3]. However, the literature on this topic is scarce.

The aim of this study was to determine the short- and mid-term prognosis in patients with calcified aortoostial coronary artery lesions who underwent RA followed by stent implantation, and to assess the feasibility and safety of the technique for this type of lesions.

## 2. Materials and Methods

### 2.1. Demographic and Clinical Data

All consecutive patients admitted in the Cardiology Department of Toulouse University Hospital who underwent angioplasty with RA during four consecutive years were retrospectively analyzed, regardless of indication for RA, and clinical data were recorded in a registry.

### 2.2. Angioplasty Procedure

Indications for myocardial revascularization included [4] acute coronary syndrome, stable coronary artery disease with myocardial ischemia confirmed by imaging (*i.e.,* >10% of myocardial area affected, >50% diameter stenosis of the left main coronary artery (LMCA), >50% diameter stenosis of the proximal left anterior descending artery (LAD)), decreased (<35%) left ventricular ejection fraction (LVEF) with positive myocardial viability assessment, or preoperative assessment.

All angioplasty procedures were performed by experienced operators in our interventional cardiology center. Pretreatment medication (aspirin and a P2Y12 receptor inhibitor) was administered to all patients; heparin was administered in order to maintain ACT > 300s.

The administration of GpIIb/IIIa inhibitors and the use of a temporary pacemaker lead or intra-aortic balloon pump counterpulsation was left to the discretion of the operator. The arterial access site was chosen according to operator preference and anatomical availability. RA was attempted either as a first procedure or following the failure of conventional PCI, according to the strategies of the operators. After engaging the artery with a guiding catheter, a 0.09-inch RA guidewire was advanced, either directly or following exchange using a microcatheter or coaxial balloon. RA was performed with a Rotablator™ system with a burr rotation speed of 140,000-200,000 rpm. The burr size was selected in order to obtain a burr-to-artery ratio <0.7, and burr runs were short. The procedure was completed by balloon dilatation and stent implantation. The use of intravascular ultrasound (IVUS), which is not reimbursed by the French National Health System, was not performed in our daily practice for such PCI. The choice of the stent was at the discretion of the operator performing the intervention. Back then, according to the French national health insurance regulations, bare metal stents are used for lesions shorter than 15 mm, artery diameters smaller than 3 mm, and nondiabetic patient.

### 2.3. Data Collection and Definitions

Patients data recorded in our registry were compiled in a specific, anonymized database.

Clinical and paraclinical data were collected from discharge letters and medical correspondences registered in the electronic patient care reporting system. Data related to revascularization procedures were obtained from interventional reports. The angiographic recording of every procedure was systematically analyzed by an interventional cardiologist in order to confirm data found in operation reports.

The initial clinical presentation included stable chronic angina, acute coronary syndrome (ACS) with/without ST segment elevation, heart failure, and preoperative assessment of patients with valvular heart disease. The left ventricular ejection fraction (LVEF) was assessed by either ultrasound, cardiac ventriculography, magnetic resonance imaging, or isotopic ventriculography. The type of platelet ADP P2Y12 receptor inhibitor that was administered to the patient was left to the operator's discretion and was also included in the registry. The duration of the dual antiplatelet therapy was 6 months, except in case of acute coronary syndrome, where it was administered for 12 months.

Aortoostial lesions were defined according to the SYNTAX score definition as a >70% stenosis of the ostial right coronary artery (RCA), or a > 50% lumen narrowing of the ostial LMCA, located within 3 mm of the coronary vessel origin. A chronic total coronary occlusion was defined as an occlusive coronary lesion in progression for over 3 months [5].

#### 2.3.1. Angioplasty Procedural Characteristics

The primary endpoint was the procedural success rate. Secondary endpoints were the rates of major adverse cardiovascular events (MACE) at 30 days and 24 months. MACE are defined by death, myocardial infarction, and stent thrombosis. Procedural success was defined according to the ROTAXUS trial by the insertion and expansion of a stent resulting in less than 20% of residual stenosis and a TIMI 3 flow at the end of the procedure [6]. Procedural complications, such as coronary artery dissection resulting in lumen occlusion (occlusive dissection) and myocardial infarction (MI), type III coronary artery perforation [7], and no/slow flow or burr entrapment, were registered in the database.

For clarity purposes, the occurrence of death, MI, or stent restenosis within 30 days following angioplasty procedure was retrospectively qualified as early major cardiac events.

The occurrence of a MI as a consequence of a coronary angioplasty was defined, according to the 2012 European recommendations, by a significant troponin elevation associated with symptoms or cardiac electrical alterations suggesting myocardial ischemia, the loss of a coronary vessel or a side branch, or myocardial necrosis confirmed with cardiac imaging. A significant troponin elevation was identified by a level of serum troponin > 5 times the upper reference limit, or by a >20% increase in the initial troponin level. The occurrence of stent restenosis was defined according to the recommendations of the Academic Research Consortium (ARC) [8].

A 2-year follow-up was completed by phone calls between August 2015 and March 2016. We also gathered information from medical reports of patients' general practitioners and referring cardiologist.

For data analysis, and according to the ARC consensus, major adverse cardiovascular events (MACE) were defined as the occurrence of death, MI, revascularization of a target lesion or a target vessel, or an ischemic stroke within 30 days up to 24 months following the angioplasty procedure.

### 2.4. Statistical Analysis

Statistical analyses were performed with Stata/SE software, version 10.1 (Stata Corp LP, College Station, USA). Categorical variables were presented as counts and percentages, while continuous variables were presented as mean ± standard deviation. Categorical data were analyzed with either a Chi2 test or Fisher's exact test, and a Student's t-test was used for continuous data. Correlated factors related to procedural success in aortoostial rotational atherectomy were studied using logistic regression. The analysis of early events, at 30 days, was based on the occurrence of death or MI, while the analysis of events at 2 years also included target lesion or vessel revascularization. The statistical significance threshold was set at 5% (p<0.05) for all tests.

Survival analyses were performed using the Kaplan–Meier estimate. Associations between the patients' characteristics of the subjects and outcomes occurrence were estimated using Cox proportional hazards regression. Multivariate analyses were conducted using multivariate Cox proportional hazards regression, following a stepwise descending method. Variables related to the considered outcome with p<0.15 in univariate analysis were taken into account in multivariate analyses.

## 3. Results

### 3.1. Clinical Characteristics

During the study period, 498 consecutive patients underwent a RA angioplasty, and procedural data were collected in our registry. Among these, 80 patients (16.1%) were treated for at least one aortoostial lesion.

The clinical characteristics of patients with and without calcified aortoostial coronary artery lesions are presented in Table 1. Patients with an aortoostial lesion who underwent RA were older ((78 ± 8 years* vs* 75 ± 10 years, p<0.001), mostly women (46.3%* vs* 26.3%, p<0.001), and presented with a lower prevalence of dyslipidemia (41.3%* vs* 59.3%, p=0.003). The average left ventricular ejection fraction (LVEF) was 48 ± 11%.

Within both groups, we noticed a high prevalence of cardiovascular risk factors and a high proportion of patients presenting with cardiovascular history. The prevalence of chronic kidney disease was high but similar in patients with and without aortoostial lesions (26.3%* vs* 24.4%, p=0.73), as one could expect based on epidemiological data of angioplasty procedures performed by rotational atherectomy. Within both groups, almost one third of patients was admitted for a stable coronary heart disease; half of them were suffering from ACS while the other half was admitted for heart failure or as part of a preoperative assessment. Dual antiplatelet therapy was administered to all patients before the rotational atherectomy procedure. The type of P2Y12 receptor inhibitor, mostly clopidogrel, was similar in both groups (83.3% in the aortoostial group* vs *84.4% in the nonaortoostial group, p=0.31).

Regarding the type of P2Y12 receptor inhibitor, clopidogrel, ticagrelor, or prasugrel, administered to the patients, results were similar in both groups: clopidogrel (83.3% in the aortoostial group* vs *84.4% in the nonaortoostial group), ticagrelor (6,8% vs 2.6%), and prasugrel (10,2% vs13%), p=0,32.

### 3.2. Angiographic and Procedural Characteristics

The angiographic characteristics of the population are presented in Table 2.

Most patients included in this study presented with a multivessel coronary artery disease. There were no statistical differences in the percentage of patients with triple-vessel disease in both groups (53.8% in the aortoostial group* vs* 46.2% in the nonaortoostial group, p=0.12). In the aortoostial group, the rotational atherectomy procedure was performed in 39 patients with a stenosis of the LMCA and in 46 patients with a stenosis of the RCA. Lesions with severe calcifications were found more frequently in patients with aortoostial lesions (76.3%* vs* 62.2%, p=0.02).

No differences were found between both groups in the rates of in-stent restenosis, chronical occlusions, or >20 mm long lesions. The amount of lesions treated with RA as a second-intention procedure following the failure of balloon angioplasty was lower in the aortoostial group (10.0%* vs* 27.5%, p=0.001). Detailed procedural characteristics are presented in Table 3. Patients with aortoostial lesions were treated less frequently through radial access (41.3%* vs *57.4%, p=0.017), and assistance devices such as intra-aortic balloon pump or temporary pacing lead were used significantly more frequently in this group (40.0%* vs* 17.5%, p<0.001).

The requirement for more than one burr during the RA procedure was more frequent in the aortoostial group (62.5%* vs* 52.4%, p=0.05); however, the burr-to-artery ratio was significantly lower (0.43 ± 0.08* vs* 0.46 ± 0.08, p=0.002). In the aortoostial group, the average number of implanted stents per lesion was 1.33 ± 0.57.

In most cases, the procedure involved the insertion of drug-eluting stents (71.3% for ostial lesions and 80.2% for nonostial lesions). The procedural success rate was high and similar in patients with and without ostial lesions (96.3%* vs *94.7%, p=0.78).

### 3.3. In-Hospital MACE and Short-Term (30 Days) Prognosis

In-hospital and 30-day clinical event rates are detailed in Table 4. The procedural complications rate was similar between both groups (7.5% in the aortoostial group* vs *8.4% in the nonaortoostial group, p=0.80). An early mortality rate, at 3 days, was observed in both groups but was significantly higher in the aortoostial group (7.5%* vs *3.4%, p=0.03). The cardiovascular complication rate at 30 days was high in both groups, with a tendency to be higher, although nonsignificantly, in the aortoostial group (13.8%* vs *7.7%, p=0.08). However, the mortality rate at 30 days was significantly higher in the aortoostial group (11.3%* vs* 4.8%, p=0.04). In multivariate analysis, the aortoostial location of the lesion treated with RA was not a factor associated with cardiovascular events at one month (HR = 1.83, 95% IC, 0.92-3.63).

### 3.4. Mid-Term (24 Months) Prognosis

A 24-month follow-up was completed for 93% of patients. Patients included in the aortoostial group showed a significantly higher rate of MACE at two-year follow-up (43.8%* vs *31.8%, p=0.04), but the mortality rate was not significantly higher in this group (30%* vs *21.8%, p=0.11) (Table 5).

**Figure 1:**
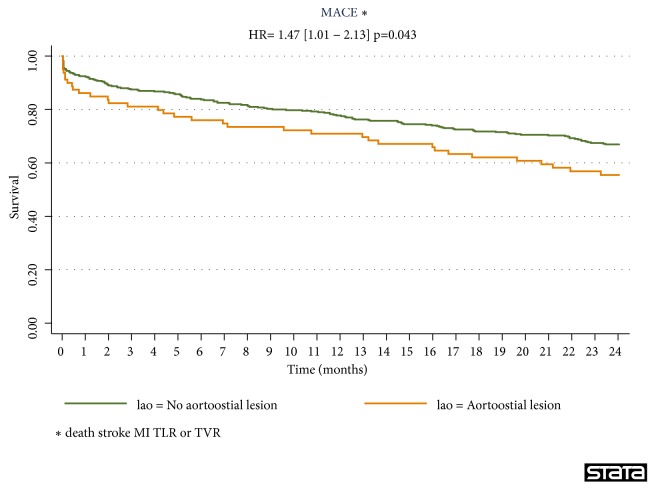
MACE-free survival at 24 months. MACE: major cardiac events; HR: hazard ratio; MI: myocardial infarction; TLR: target lesion revascularization; TVR: target vessel revascularization.

In multivariate analysis, we observed that the variables associated with the occurrence of MACE at 24 months were the RA procedure of an aortoostial lesion (RR=1.52, 95% IC, 1.03-2.26, p=0.035), the age (RR=1.20, 95% IC, 1.01-1.43, p=0.035), hemodialysis (RR=4.13, 95% IC, 2.10-8.13, p<0.001), a history of lower extremity peripheral artery disease (RR=1.63, 95% IC, 1.16-2.30, p=0.005), and the left ventricular ejection fraction (LVEF<30%; HR=3.32, 95% IC; 1.77-6.23; p<0.001) (Table 6). A multivessel coronary artery disease and the location of the treated coronary stenosis on the distal LMCA are not associated with cardiovascular events at 24 months. In multivariate analysis, the aortoostial location of the lesion treated with RA was an independent predictive factor of cardiovascular events at 24 months (Figure 1).

## 4. Discussion

Percutaneous intervention of ostial lesions in coronary arteries is technically challenging. Our results show a poor short-term and mid-term prognosis in patients treated with RA for an aortoostial coronary lesion. Indeed, these patients showed a higher mortality rate at 30 days and a higher cardiovascular complications rate at 24 months than those observed for the rest of the population treated with rotational atherectomy in our center. In particular, the aortoostial location of the lesion increased by 1.5 times the risk of cardiovascular events at 24 months. This increase in the 30-day mortality rate cannot be explained by the incidence of procedural complications, as they were similar in both groups.

An increase in MACE rate following PCI of calcified lesions was described in previous publications [3, 9, 10]. Aortoostial lesions are rare, but they remain a technical challenge, in particular because of a higher prevalence of calcified lesions, turbulent blood flow, elastic recoil of the artery, and a more complicated stent implantation, as opposed to nonaortoostial lesions. Moreover, aortoostial lesions are part of an aortic wall pathology that is not limited to the coronary artery in its ostium; assessing the size of the artery and lesion prior to stenting can be difficult. The rate of restenosis in ostial lesions is higher than in nonostial lesions. The aggressive, balloon-mediated, dilatation of aortoostial lesions may cause retrograde dissections of the coronary ostium up to the ascending aorta. Some factors, specific to the angioplasty of aortoostial lesions, may explain these higher rates of restenosis. Indeed, a good visibility of the coronary ostium is sometimes difficult to achieve during stent implantation. Stent under expansion, longitudinal compression of the stent in the ostium, and incomplete ostial coverage may occur, resulting in restenosis. As a result, the projection must be carefully adjusted during stent placement in order to obtain a good ostial visibility. Heavy calcification, more frequent in aortoostial lesions, may be responsible for the misplacement of a stent, thus facilitating stent thrombosis. At last, the question arises as to whether the radial force of last-generation, drug-eluting stents, with thinner struts, is strong enough to prevent the elastic recoil of the artery to its aortoostial segment. Patel et al. [11] showed, in a monocentric and retrospective study, with younger patients, that the use of IVUS for PCI of coronary ostial lesions was associated with significant lower rates of MACE (19% vs 38%, p: 0,004). The use of IVUS was not assessed in our study as it is not reimbursed in our country and is thus not performed in our daily practice for such PCI. We thus cannot assess the impact it might have on our results.

This poor short-term and mid-term prognosis in patients with aortoostial coronary lesions who underwent RA may be due to their older age, comorbidities, and a high cardiovascular risk profile. Indeed, it was clearly demonstrated that the presence of calcification is an independent risk factor of cardiovascular events [12, 13] and that it increases the complication risk following balloon angioplasty [14]. Moreover, noncompliant, calcified plaques often require high pressure dilatation of the artery prior to stent implantation, hence increasing the risk for coronary dissections and thrombosis. The stress applied by the balloon on the vessel may not be homogenous along the lesion, in particular due to the location of the calcifications, which increases the risk for acute occlusion of the vessel, restenosis, and MACE [15]. In particular, angiographic studies have shown that the coronary vessel lumen diameter gained following the procedure, as well as the diameter of the residual stenosis, is lower in the presence of vascular calcifications [16].

Our results also demonstrate that rotational atherectomy is a reliable procedure for treating aortoostial lesions. Indeed, the success rate of RA in patients with aortoostial lesions obtained in this study was excellent, about 96%, and similar to the success rate observed for the rest of our cohort.

Moreover, our study highlighted the procedural complications encountered after all RA procedures performed during the inclusion period, independently of the indication for the procedure. In particular, 7% of patients presented with at least one complication following RA procedure in both groups, with a tendency to a higher rate of cardiovascular events in the group of patients with aortoostial lesions. The absence of discrepancies in the amount of angiographic complications in both groups confirms the safety of RA in the treatment of aortoostial lesions. Moreover, the complications rates observed in this study are closed to those presented in previous reports on rotational atherectomy [6, 17–19].

Although this was a single-center study, it was based on the comprehensive and representative population of all patients admitted to our institution: many of these patients were addressed from various centers and some data, such as LVEF assessment, were collected locally prior to admission to our institution. The clinical and angiographic characteristics of patients treated with RA differ among other studies on the topic. In comparison with available data in the literature, our population presents more severe characteristics, in particular an older population on average and a high prevalence of patients admitted for ACS (about 50% in this study). Available data are scarce regarding RA performed in a context of ACS. In previous studies, most of the included patients underwent rotational atherectomy for a stable coronary heart disease. Moreover, we also observed a high frequency of severe coronary diseases in our population, with almost half of the patients suffering from triple-vessel disease. The severity of our patients' condition and baseline comorbidities may partially explain the high rate of MACE at 24-month follow-up.

## 5. Limitations

All percutaneous coronary interventions were carried out by experienced operators. The use of IVUS, which is not reimbursed in France, should be tested in such PCI with RA in ostial lesion in order to assess any potential improvement in stent sizing and apposition.

However, the requirements of angioplasty followed by RA stenting procedures involve that these can only be carried out in a limited number of high-volume centers, with specific facilities and trained operators, such as those available in our institution. Patients with calcified aortoostial lesions represented the minority of patients included in our registry, thus hampering the performance of meaningful statistical comparisons. Moreover, the long-term outcomes of rotational atherectomy in calcified ostial lesions are unknown. As this was a retrospective analysis, some data were thus unavailable, but the 2-year follow-up was completed for 93% of the patients.

Other limitations include the fact that data about the presence of aortic valvular stenosis and its severity were not collected in this study, although this parameter could have been evaluated, and that the SYNTAX score was not assessed for this cohort of patients with calcified lesions.

## 6. Conclusion

Treatment of aortoostial lesions in the coronary arteries is technically challenging. Our study included 498 consecutive patients treated with rotational atherectomy and its results confirmed a worse short-term and mid-term prognosis in patients with an aortoostial calcified coronary stenosis: these patients showed a higher mortality rate at 30 days and a higher cardiovascular complications rate at 24 months. Moreover, this study also showed that the aortoostial location of the lesion treated with RA was an independent predictive factor of cardiovascular events at 24 months.

Nevertheless, our results also demonstrated that rotational atherectomy was a feasible and safe procedure for treating aortoostial lesions. Indeed, the success rate of RA procedure for treating aortoostial lesions was excellent and the complication rate was similar to the rate observed in the rest of the population. Further studies with long-term follow-up are required in order to determine the optimal strategy for calcified aortoostial lesions.

## Figures and Tables

**Table 1:** Baseline demographic characteristics.

	Aortoostial lesions n=80 (%)	Nonostial lesions n=418 (%)	p-value
Age (years)	78 ± 8	75 ± 10	<0.01

Female	37 (46.3)	110 (26.3)	<0.001

Active smoking	14 (17.5)	73 (17.5)	0.99

Diabetes	35 (43.8)	162 (38.8)	0.40

Dyslipidemia	33 (41.3)	248 (59.3)	0.003

BMI (kg/m^2^)	26.1 ± 4.5	26.7 ± 4.6	0.35

Family history of CVD	5 (6.3)	52 (12.4)	0.11

Coronary angioplasty	23 (28.8)	122 (29.3)	0.92

Coronary artery bypass grafting	10 (12.5)	52 (12.5)	0.99

Stroke	7 (8.8)	36 (8.6)	0.97

Lower extremity peripheral artery disease	21 (26.6)	95 (22.8)	0.47

History of coronary artery disease	35 (43.8)	191 (45.8)	0.74

LVEF on admission (%)	48 ± 11	48 ± 12	0.75

Hemodialysis	1 (1.3 %)	14 (3.3 %)	0.47

*Clinical presentation*			

Stable coronary artery disease	24 (30.0)	140 (33.5)	0.75

Acute coronary syndrome	40 (50.0)	206 (49.3)	0.75

Chronic heart failure	11 (13.8)	42 (10.0)	0.75

Preoperative assessment	5 (6.3)	30 (7.2)	0.75

BMI: body mass index; CVD: cardiovascular disease; LVEF: left ventricular ejection fraction.

**Table 2:** Angiographic characteristics of the population.

	Aortoostial lesions n=80 (%)	Nonostial lesions n=418 (%)	p-value
*CAD extension*			

Multivessel CAD	63 (78.8)	347 (83.0)	0.12

*Lesion location*			

LMCA	39 (48.8)	125 (29.9)	<0.001

Right coronary artery	46 (57.5)	118 (28.2)	<0.001

LAD	-	211 (50.0)	<0.001

Circumflex artery	-	57 (13.6)	0.03

*Characteristics*			

In-stent restenosis	0 (0.0)	19 (4.5)	0.06

Chronic total occlusion	4 (5.0)	38 (9.1)	0.23

Severe calcification	61 (76.3)	260 (62.2)	0.02

Lesion length >20 mm	38 (47.5)	245 (58.6)	0.07

CAD: coronary artery disease; LMCA: left main coronary artery; LAD: left anterior descending artery.

**Table 3:** Procedural characteristics.

	Aortoostial lesions n=80 (%)	Nonostial lesions n=418 (%)	p-value
Radial approach	33 (41.3)	240 (57.4)	0.017

Guide catheter size			

6F	34 (42.5)	181 (43.9)	0.95

7F	43 (53.8)	212 (51.5)	0.95

Procedure duration (min)	83 ± 50	87± 46	0.51

Mean contrast medium volume (ml)	194 ± 92	213 ± 94	0.10

> 1 burr requirement	50 (62.5)	219 (52.4)	0.05

Burr to artery ratio	0.43 ± 0.08	0.46 ± 0.08	0.002

Number of stents/lesions	1.33 ± 0.57	1.37 ± 0.58	0.49

Stent*∗*			0,07
DES only	57 (71,3%)	328 (80,2%)	
DES or bare metal stent	23 (28,8%)	81 (19,2%)	

Procedural success rate	77 (96.3)	396 (94.7)	0.78

(*∗*) missing data for 9 patients.

**Table 4:** In-hospital and 30-day clinical event rates.

	Aortoostial lesions n=80 (%)	Nonostial lesions n=418 (%)	p-value
Angiographic complications	6 (7.5)	35 (8.4)	0,8

Burr entrapment	0 (0.0)	2 (0.5)	1.00

Perforation	2 (2.5)	9 (2.2)	0.69

No/slow flow	2 (2.5)	17 (4.1)	0.75

Occlusive dissection	1 (1.3)	4 (1.0)	0.59

3-day mortality rate	6 (7.5)	10 (2.4)	0.03

Cardiac tamponade	2 (2.5)	7 (1.7)	0.64

30-day mortality rate	9 (11.3)	20 (4.8)	0.04

Myocardial infarction (MI)	6 (7.5)	13 (3.1)	0.10

Vascular complications	8 (10.0)	28 (6.7)	0.30

30-day event rate (death and MI)	11 (13.8)	32 (7.7)	0.08

**Table 5:** MACE at 24 months.

	Aortoostial lesions n=80 (%)	Nonostial lesions n=418 (%)	p-value
MACE	35 (43.8)	133 (31.8)	0.04

24-month mortality rate	24 (30)	91 (21.8)	0.11

Myocardial infarction	11 (13.8)	39 (9.3)	0.23

TLR/TVR	10 (12.5)	27 (6.5)	0.06

Stroke	0 (0)	1 (0.2)	1.00

TLR: target lesion revascularization; TVF: target vessel revascularization.

**Table 6:** Factors associated with 24-month MACE, multivariate analysis.

Variables	HR	95% CI	p-value
Aortoostial lesions	1.52	1.03-2.26	0.035

Age (10-year gain)	1.20	1.01-1.43	0.035

Hemodialysis	4.13	2.10-8.13	<0.001

History of lower extremity PAD	1.63	1.16-2.30	0.005

LVEF <30%	3.32	1.77-6.23	<0.001

Artery diameter > 3mm	0.57	0.40-0.82	0.028

PAD: peripheral artery disease; LVEF: left ventricular ejection fraction.

## Data Availability

The data used to support the findings of this study are available from the corresponding author upon request.

## Abbreviations

ACS: Acute coronary syndrome
LAD: Left anterior descending artery
LMCA: Left main coronary artery
LVEF: Left ventricular ejection fraction
MACE: Major adverse cardiovascular events
MI: Myocardial infarction
RA: Rotational atherectomy
RCA: Right coronary artery.
